# Porcine Circovirus Type 3 Enters Into PK15 Cells Through Clathrin- and Dynamin-2-Mediated Endocytosis in a Rab5/Rab7 and pH-Dependent Fashion

**DOI:** 10.3389/fmicb.2021.636307

**Published:** 2021-02-16

**Authors:** Ruihan Shi, Lei Hou, Li Wei, Rong Quan, Bin Zhou, Haijun Jiang, Jing Wang, Shanshan Zhu, Jiangwei Song, Dan Wang, Jue Liu

**Affiliations:** ^1^Beijing Key Laboratory for Prevention and Control of Infectious Diseases in Livestock and Poultry, Institute of Animal Husbandry and Veterinary Medicine, Beijing Academy of Agriculture and Forestry Sciences, Beijing, China; ^2^College of Veterinary Medicine, Yangzhou University, Yangzhou, China; ^3^Jiangsu Co-Innovation Center for Prevention and Control of Important Animal Infectious Diseases and Zoonoses, Yangzhou University, Yangzhou, China; ^4^College of Veterinary Medicine, Nanjing Agricultural University, Nanjing, China

**Keywords:** PCV3, PK15 cells, replication, endocytic pathway, endosomal trafficking

## Abstract

Porcine circovirus type 3 (PCV3) invades multiple tissues and organs of pigs of different ages and are widely spread throughout pig farms, emerging as an important viral pathogen that can potentially damage the pig industry worldwide. Since PCV3 is a newly discovered virus, many aspects of its life cycle remain unknown. Porcine kidney epithelial cells are important host targets for PCV3. Here, we used systematic approaches to dissect the molecular mechanisms underlying the cell entry and intracellular trafficking of PCV3 in PK15 cells, a cell line of porcine kidney epithelial origin. A large number of PCV3 viral particles were found to colocalize with clathrin but not caveolin-1 after entry, and PCV3 infection was significantly decreased when treated with chlorpromazine, dynasore, knockdown of clathrin heavy chain expression via RNA interference, or overexpression of a dominant-negative mutant of EPS15 in PCV3-infected cells. After internalization, the viral particles were further observed to colocalize with Rab5 and Rab7, and knockdown of both expression by RNA interference significantly inhibited PCV3 replication. We also found that PCV3 infection was impeded by ammonium chloride treatment, which indicated the requirement of an acidic environment for viral entry. Taken together, our findings demonstrate that PCV3 enters PK15 cells through a clathrin- and dynamin-2-mediated endocytic pathway, which requires early and late endosomal trafficking, as well as an acidic environment, providing an insightful theoretical basis for further understanding the PCV3 life cycle and its pathogenesis.

## Introduction

Porcine circovirus (PCV), classified into the genus *Circovirus* of the family *Circoviridae*, is a non-enveloped, single-stranded circular DNA virus (Tischer et al., 1986; Ellis, 2014). PCV1 is non-pathogenic to pigs (Cheung, 2003); however, PCV2 is widely acknowledged as a major pathogen for porcine circovirus-associated disease (PCVAD), leading to large economic losses for the swine industry worldwide. Porcine dermatitis and nephropathy syndrome (PDNS) are recognized as important components of PCVAD, and the incidence of PDNS may exceed that of porcine post-weaning multisystemic wasting syndrome in Europe and the United Kingdom (Gresham et al., 2000; Rose et al., 2012; Opriessnig and Langohr, 2013). In 2015, a novel circovirus, designated PCV3, was shown to play a potential etiological role in PDNS (Palinski et al., 2017).

The PCV3 genome is composed of approximately 2,000 nucleotides, considerably larger than that of PCV2 (approximately 1,780 nucleotides) (Zhai et al., 2014), and contains three predicted open-reading frames (ORFs), including ORF1, ORF2, and ORF3, which encode the replicase (rep), and capsid (cap) protein, as well as a protein of unknown function, respectively (Palinski et al., 2017). PCV3 was first identified based on metagenomic sequencing analysis in PCV2-negative sows with acute death, PDNS-like clinical manifestations, and reproductive failure in the United States (Palinski et al., 2017). Immunohistochemical (IHC) tests showed that the PCV3 antigen colocalized with typical PDNS histopathological lesion sites, and a retrospective study demonstrated that IHC analysis yielded a 93.8% positive rate for PCV3 infection in PCV2-negative cases with PDNS histopathological lesions (Palinski et al., 2017). Efforts to experimentally reproduce PDNS with PCV2 have failed, whereas Jiang et al. successfully reproduced PDNS-like disease by intranasally inoculating pathogen-free piglets with PCV3 rescued from an infectious PCV3 genome clone (Krakowka et al., 2008; Palinski et al., 2017; Jiang et al., 2019), strongly supporting the hypothesis that PCV3 plays a potentially etiologic role in PDNS lesions. In addition to PDNS, PCV3 was reported to be associated with congenital tremors, reproductive failure, and multi-systemic inflammation (Phan et al., 2016; Chen et al., 2017; Palinski et al., 2017).

Cell entry is the first and most essential stage of viral infection. Many viruses can exploit cellular endocytic pathways, including clathrin-mediated endocytosis (CME), caveolar/lipid-raft-mediated endocytosis (CvME), macropinocytosis, and clathrin- and caveolar-independent endocytosis (CCIP), to gain access to host cells (Sieczkarski and Whittaker, 2002a; Mercer et al., 2010; Helenius, 2018). Both CME and CvME are the most commonly observed endocytosis pathways utilized by viruses to gain access to host cells (Mercer et al., 2010). Clathrin and caveolin-1 are important structural proteins and labels for clathrin- and caveolar-dependent endocytic vesicles, respectively (Takei and Haucke, 2001; Pelkmans and Helenius, 2002). The work of Misinzo et al. (2005) showed that PCV2 uses a CME pathway to gain entry into 3D4/31 cells, a monocytic cell line, and that an acidic environment was required for its successful infection. In porcine epithelial cells, the CME pathway provides a non-reproductive entry route for PCV2, where the virus is allowed to internalize and fulfill successful infection via a pathway independent of clathrin, caveolae, or dynamin, but requires actin and small GTPases (Misinzo et al., 2009). For PCV3, the epithelial cells, especially the kidney epithelial cells also serve as important host targets. In pigs experimentally infected with PCV3, strong positive signals for the PCV3 antigen were observed in kidney epithelial cells by immunohistochemistry assay, where the intensity of the positive signal was proportional to the degree of pathological damage (Jiang et al., 2019). In addition, porcine kidney epithelial cells have been shown to support PCV3 isolation and propagation *in vitro* (Jiang et al., 2019; Oh and Chae, 2020). However, the underlying mechanisms of PCV3 internalization in porcine kidney epithelial cells remain obscure. Although both belong to the porcine circovirus, major differences were established between PCV2 and PCV3 in terms of genome structure and clinical manifestations. Plentiful eosinophil infiltrations presented in the lymphoid tissues of PCV3-infected pigs were speculated to mediate a more severe allergic reaction than PCV2 infection, which may eventually trigger clinical signs such as skin rashes and asthma (Jiang et al., 2019), whereas in PCV2-infected pigs, an increase in circulating neutrophils rather than monocytes, basophils, or eosinophils was characterized 7–14 days post-infection (Gauger et al., 2011). The similarities of the cap and rep proteins between PCV2 and PCV3 are only 37 and 55%, respectively (Palinski et al., 2017). Previous studies showed that glycosaminoglycans (GAGs), including heparan sulfate and CS-B, were shown to be attachment receptors for PCV2 (Misinzo et al., 2006). The conserved sequence, XBBXBX, is a heparan sulfate-binding motif (X stands for a neutral/hydrophobic amino acid; B stands for a basic amino acid) presented on the cap of PCV2 as ^98^IRKVKV^103^ (Cardin and Weintraub, 1989; Misinzo et al., 2006). However, this motif cannot be found in the cap of PCV3, owing to an alteration in the second amino acid. Accordingly, we speculate that the cell surface receptors for PCV2 and PCV3 are not the same, which may likely result in different endocytic and infection mechanisms, and may contribute to the differences noted in host immune reactions.

In this study, systematic approaches, including biochemical inhibition, confocal microscopy analysis, RNA interference, and expression of DNA mutants, were employed to dissect the molecular mechanism underlying cell entry as well as the endosomal trafficking of PCV3 in PK15 cells, and elucidate the involvement of the host factors in this process. We demonstrated that PCV3 invasion into PK15 cells involved a clathrin- and dynamin-2-dependent endocytic pathway requiring early and late endosomal trafficking and an acidic environment for efficient infection.

## Materials and Methods

### Cells, Viruses, Reagents, and Antibodies

PK15 cells (CRL-1711) free from PCVs were originally obtained from the American Type Culture Collection, which were cultivated in Dulbecco’s modified Eagle’s medium (DMEM, 11995, Life Technologies, United States) supplemented with 5% newborn calf serum (NBCS, Gibco; Life Technologies, 16010159), 0.2% NaHCO_3_, and 1% penicillin-streptomycin. The PCV3 strain rescued from an infectious PCV3 genome clone PCV3/CHN/Hebei-LY/2015 (MF318451) by Jiang et al. (2019) was used in this study. This PCV3 strain was successfully propagated in PK15 cells. Pharmacological inhibitors, including genistein, methyl-beta-cyclodextrin (M-β-CD), amiloride, and ammonium chloride (NH_4_Cl), were obtained from Sigma-Aldrich; cytochalasin D (Cyto D) was purchased from Abcam; chlorpromazine (CPZ) and dynasore were purchased from Selleck Chemicals. Alexa Fluor 647-conjugated cholera toxin subunit B (CT-B, C-34778) was purchased from Invitrogen.

Rabbit anti-Rab5A (11947-1-AP), rabbit anti-Rab7A (55469-1-AP), rabbit anti-Rab11A/B (15903-1-AP), and mouse anti-clathrin heavy chain (CLTC) (66487-1-Ig) primary antibodies were purchased from Proteintech (Chicago, United States). Rabbit anti-caveolin-1 (3238) primary antibody was purchased from Cell Signaling Technology (Boston, MA, United States). Mouse anti-β-actin (A5441) primary antibody, tetramethyl rhodamine isothiocyanate (TRITC)-conjugated goat anti-rabbit (T6778), TRITC-conjugated goat anti-swine (SAB3700424), TRITC-conjugated goat anti-mouse (T5393), and fluorescein isothiocyanate (FITC)-conjugated goat anti-swine (SAB3700433) antibodies were obtained from Sigma-Aldrich (Saint Louis, United States). Swine polyclonal antibody against the PCV3 Cap prepared in our laboratory was used in immunofluorescence assays, and a mouse monoclonal antibody against the PCV3 Cap prepared in our laboratory was employed in western blotting.

### Inhibitor Pretreatment and Virus Infection

Cells were seeded in chamber slides and exposed to the indicated concentrations of inhibitors and dimethyl sulfoxide (DMSO) (mock) or ddH_2_O (mock) for 1 h at 37°C, and then infected with PCV3 (4.5 log_10_ TCID_50_/0.1 ml) at a multiplicity of infection (MOI) of 0.5 in the presence of the inhibitors. After infection for 1 h at 37°C, the cultured cells were incubated in a medium supplemented with the indicated concentrations of CPZ, dynasore, genistein, M-β-CD, amiloride, and NH_4_Cl for 48 h, fixed, stained with PCV3 Cap antibodies, and visualized by immunofluorescence microscopy. Five images from each sample were randomly captured from various regions, each with more than 50 cells. The proportion of PCV3-infected cells was determined by ImageJ analysis, and the data were analyzed using the statistical analysis software GraphPad Prism. Alternatively, at 48 h post infection (hpi), the cells were collected and subjected to western blot assay targeting PCV3 cap. The experiments were conducted in triplicate, and the error bars represent the standard deviations.

### Cell Viability Assays

Cell viability upon inhibitor treatment was evaluated using a 3-(4, 5-dimethylthiazolyl-2)-2, 5-diphenyltetrazolium bromide (MTT) kit (M1020, Solarbio, Beijing, China) in accordance with the manufacturer’s instructions. Briefly, PK15 cells grown on 96-well plates were incubated with the indicated concentrations of CPZ, dynasore, genistein, M-β-CD, amiloride, and NH_4_Cl for 48 h, or incubated with Cyto D for 2 h at 37°C. Then, 90 μl of fresh medium and 10 μl of MTT reagent were added to the cells. After incubating for 1–4 h at 37°C, the supernatant was discarded and 110 μl of formazan reagent was added, followed by a low-speed oscillation for 10 min. The absorbance value at a wavelength of 490 nm was detected using a microplate reader (iMark, BIO-RAD). Cells incubated with DMSO (solvent for CPZ, dynasore, genistein, Cyto D, and amiloride) and ddH_2_O (solvent for M-β-CD and NH_4_Cl) served as the mock-treated groups, respectively. The working concentrations of all inhibitors used in the virus infection or CT-B uptake assays showed no significant cytotoxic effects.

### CT-B Uptake Assay

Cells were seeded in chamber slides and pretreated with the indicated concentrations of M-β-CD or genistein for 1 h at 37°C. Then 25 μg/ml CT-B-AF647 was added to the cell cultures in the presence of the inhibitors for 2 h at 37°C. Next, the cells were washed with phosphate-buffered saline (PBS) three times to remove the non-attached and non-internalized CT-B-AF647 and fixed in 4% paraformaldehyde (PFA) for 20 min. The cells were stained with 4′, 6′-diamidino-2-phenylindole (DAPI) for 5 min at room temperature (RT), mounted, and visualized by immunofluorescence microscopy.

### Immunofluorescence Assay (IFA) and Confocal Microscopy

PK15 cells growing on chamber slides were fixed with 4% PFA for 20 min. After permeabilization with 0.1% Triton X-100 for 10 min, the samples were blocked in 5% skim milk for 2 h at RT. Next, the samples were probed with the corresponding primary antibodies for 16 h at 4°C. After washing three times with PBS, the samples were incubated with the appropriate secondary antibodies for 1 h at 37°C, and then stained with DAPI for 5 min at RT. Samples were observed under a laser scanning confocal fluorescence microscope (Nikon Instruments, Inc., A1R, Japan).

### TCID_50_ Assay

The PCV3 titers in cell cultures were determined on PK15 cell monolayers by IFA, as in the case of PCV2 (Fenaux et al., 2002; Wei et al., 2008). Briefly, the viral samples were serially diluted 10-fold in serum-free DMEM and inoculated on PK15 cell monolayers. After incubating for 1 h at 37°C, fresh DMEM supplemented with 2% NBCS was added and the samples were incubated. At 72 hpi, the expression of PCV3 Cap was examined through IFA and the virus titer was assayed as TCID_50_ per 0.1 ml.

### Plasmid Transfection and RNA Interference (RNAi)

Recombinant plasmids expressing GFP-tagged wild-type (WT)-EPS15 (DIIIΔ2) and GFP-tagged DN-EPS15 (EΔ95/295) mutant proteins were generously provided by Sandrine Belouzard (Institute Pasteur de Lille, France) (Lecot et al., 2005). Recombinant plasmids expressing GFP-tagged caveolin-1-WT and GFP-tagged caveolin-1-DN were generously provided by Collin R. Parrish (Cornell University, Cornell, United States).

Cells grown to 80% confluence on 6-well plates were transfected with siRNAs using Lipofectamine RNAiMAX Transfection Reagent (Invitrogen, 13778150) according to the manufacturer’s instructions. The siRNAs targeting CLTC (siCLTC, sc-35067), caveolin-1 (siCav1, sc-29241), Rab5 (siRab5, sc-36344), Rab7 (siRab7, sc-29460), and siRNA with a control sequence (siCtrl, sc-37007) were purchased from Santa Cruz Biotechnology. The siRNA siRab11 (AM16708-184756) was purchased from Thermo Fisher Scientific. Next, 10 nM (data not shown) and 20 nM of each siRNA were transfected and assayed for silencing efficiency by western blot (WB) analysis. From this, a concentration of 20 nM was proven effective and was used in subsequent RNAi experiments. PK15 cells were transfected with siRNA for 48 h followed by infection with PCV3. At 36 h post PCV3 infection, the cells were harvested and the extracted protein was subjected to WB for viral Cap expression quantification. Alternatively, the cell cultures were collected, and the virus titer was assayed by TCID_50_ after three freeze-thaw cycles. For the plasmid transfection assay, cells grown to 60% confluence on 6-well plates were transfected with the appropriate plasmids using Lipofectamine LTX Reagent with PLUS Reagent (Invitrogen, 15338030). After transfection for 24 h, the cells were infected with PCV3. At 36 h post PCV3 infection, the cells were harvested and subjected to WB assay or fixed and subjected to IFA for confocal microscopy analysis.

### Western Blotting

Cell samples were washed twice with PBS and then lysed in RIPA lysis buffer supplemented with 1 mM phenylmethanesulfonylfluoride (PMSF) on ice for 5 min. The cell lysis samples were collected and the supernatants were isolated after centrifugation at 12,000 rpm for 20 min at 4°C. The bicinchoninic acid kit (Thermo Fisher Scientific, 23225) was used for protein concentration measurement. Equal amounts of protein were loaded for each sample and then subjected to sodium dodecyl sulfate (SDS)-polyacrylamide gel electrophoresis (PAGE) for separation. The proteins in the gel were then transferred to a nitrocellulose membrane followed by antibody-probed immunoblotting. The band density was determined by the software Image J.

### Statistical Analysis

Data were processed using GraphPad Prism software and displayed as means ± standard deviations of three independent tests. Statistical significance was determined through one-way analysis of variance, the least significant difference test and paired *t*-test. Each experiment was performed at least in triplicate, and the error bars represent the standard deviation. *^∗^P* < 0.05, *^∗∗^P* < 0.01 were considered statistically significant.

## Results

### PCV3 Particles Were Colocalized With Clathrin After Internalization

**FIGURE 1:**
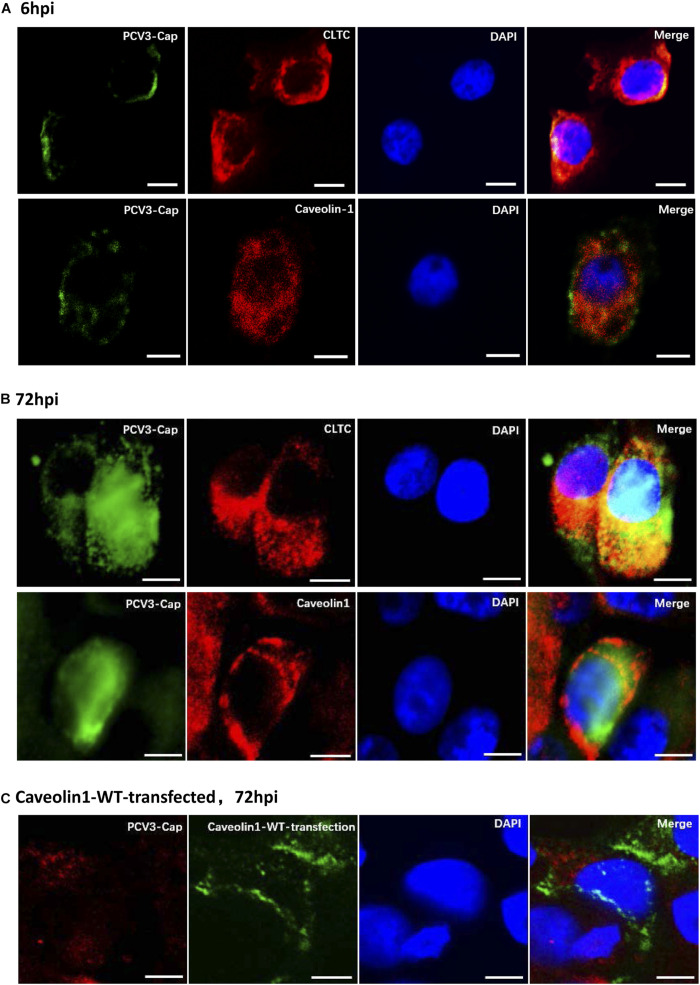
Immunofluorescence colocalization analysis of the PCV3 Cap with clathrin and caveolin-1 in PK15 cells. A large amount of PCV3 virions colocalized with clathrin after internalization. **(A,B)** PK15 cells seeded in chamber slides were incubated with PCV3 and cultured in a medium supplemented with 2% NBCS for 6 h **(A)** or 72 h **(B)** before being fixed with 4% PFA at RT for 20 min. Double immunofluorescence staining to detect the PCV3 Cap (FITC) and clathrin (TRITC) or caveolin-1 (TRITC) was performed before being examined by fluorescence confocal microscopy. **(C)** GFP-tagged caveolin-1-WT transfected cells were infected with PCV3. At 72 hpi, the cells were fixed and subjected to immunofluorescence staining to detect the PCV3 Cap (TRITC) and confocal microscopy analysis of the PCV3 Cap with exogenous expressed caveolin-1 (GFP) was performed. The yellow color indicates colocalization in the merged images. Scale bar, 10 μm.

Clathrin and caveolin-1 proteins are labels for clathrin- and caveolin-dependent endocytic vesicles, respectively. Immunofluorescence colocalization analysis was firstly conducted to analyze whether one of the both classical endocytic pathways play a role in PCV3 internalization. Our results showed that at 6 hpi, positive signals for PCV3 Cap were mainly distributed near the cell membrane and most of them were observed to colocalize with clathrin (Figure 1A). At 72 hpi, the colocalization of a large amount of PCV3 Cap proteins with clathrin was clearly observed, indicating the continuous entry of PCV3 into PK15 cells in a clathrin-mediated manner (Figure 1B). However, double immunofluorescence staining showed that colocalization of the virions with caveolin-1 was almost absent either near the cell membrane or in the cytoplasm at 6 and 72 hpi (Figures 1A,B). In addition, no obvious colocalization between PCV3 Cap and exogenously expressed caveolin-1 was observed in green fluorescent protein (GFP)-tagged WT transfected PK15 cells (Figure 1C). These findings demonstrated that the entry of PCV3 into PK15 cells was associated with clathrin but not caveolae.

### Inhibition of Clathrin-Mediated Endocytosis Reduced PCV3 Infection

**FIGURE 2:**
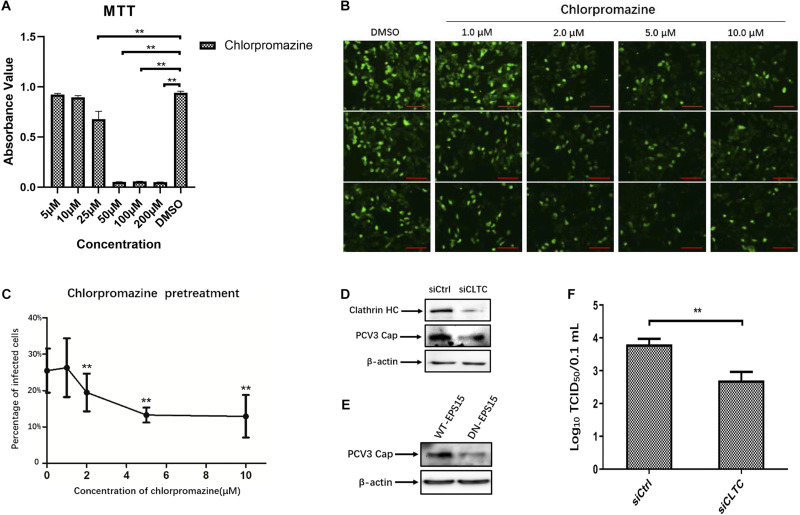
Clathrin-mediated endocytosis is involved in PCV3 infection. **(A)** Cell viability upon CPZ treatment was evaluated by MTT assay. **P < 0.01 indicates statistical significance when compared with the DMSO-treated group. **(B,C)** Chlorpromazine inhibited PCV3 infection. PK15 cells were exposed to CPZ at the indicated doses or DMSO (mock) for 1 h, followed by PCV3 infection for 1 h. After culturing in a medium supplemented with CPZ and 2% NBCS for 48 h, the cells were probed using anti-PCV3 Cap antibodies and observed under immunofluorescence microscopy. Scale bar, 50 μm. The infection rate of PCV3 in PK15 cells decreased after CPZ pretreatment in a dose-dependent pattern, as determined by the percentage of infected cells. The vertical three pictures were taken from the same cell groups treated with the indicating concentrations of CPZ. **P < 0.01 indicate statistical significance when compared with the mock-treated group (0 μM). **(D)** Cells were transfected with 20 nM siCtrl or 20 nM siCLTC followed by PCV3 infection. After incubated in a medium supplemented with 2% NBCS for 36 h, the total cell protein was extracted and analyzed by WB targeting PCV3-Cap and CLTC to determine the knockdown efficiency and viral Cap expression. β-actin served as the loading control for protein detection. **(E)** EPS15-WT or EPS15-DN transfected cells were exposed to PCV3 and incubated in a medium supplemented with 2% NBCS for 36 h. The extracted protein was examined by WB with anti-Cap antibodies to determine viral Cap expression. β-actin served as the loading control for protein detection. **(F)** PK15 cells transfected with 20 nM siCtrl or 20 nM siCLTC were infected with PCV3 and incubated in medium supplemented with 2% NBCS for 36 h. Thereafter, the cell cultures were harvested, and the viral titer were assessed by TCID_50_ analysis after three freeze-thaw cycles. **P < 0.01 indicated statistical significance compared with the siCtrl groups.

The observed colocalization of the PCV3 Cap with clathrin suggests that CME may be involved during PCV3 infection in PK15 cells. Therefore, we further used pharmacological perturbations combined with RNA interference and expression of DN mutant proteins to verify the findings and better characterize the involvement of clathrin in PCV3 infection. CPZ is a commonly used drug inhibitor that specifically blocks the CME pathway by impeding clathrin lattice polymerization (Wang et al., 1993). In this study, CPZ treatment was performed on PK15 cells prior to and during PCV3 infection to investigate its impact on viral endocytosis. The working concentrations of CPZ were determined according to the MTT assays for cell viability analysis, and the concentrations of CPZ used in this experiment showed no significant cytotoxic effects (Figure 2A). We observed that the proportion of PCV3-infected cells decreased significantly after CPZ pretreatment in a dose-dependent manner, as indicated by IFA assays probing the PCV3 Cap (Figures 2B,C). Pretreatment of cells with 2, 5, and 10 μM of CPZ reduced PCV3 infection from 25.5% in the untreated cells to 19.5, 13.3, and 12.9%, respectively (Figures 2B,C). The involvement of clathrin in PCV3 infection was further evaluated by RNA interference targeting CLTC. PK15 cells transfected with 20 nM siCLTC were infected with PCV3 followed by 36 h incubation. The level of PCV3 Cap expression was determined by WB and the viral titers were assayed by TCID_50_. Western blot analysis demonstrated that the expression of PCV3 Cap was downregulated in siCLTC-transfected cells inoculated with PCV3 compared to cells pre-transfected with control siRNA (siCtrl) (Figure 2D). Accordingly, the virus titers in the cell cultures decreased significantly after siCLTC transfection compared with the control siRNA-transfected groups (Figure 2F). In addition, we transfected the WT or the DN constructs of EPS15, one of the adaptors involved in CME, into PK15 cells prior to PCV3 infection to determine the effects of overexpression upon viral infection (Benmerah et al., 1999; Popova et al., 2013). The results showed that the level of PCV3 Cap protein expression in EPS15-DN transfected cells was lower than that in EPS15-WT transfected cells, as determined by WB analysis (Figure 2E). These results provide strong evidence that CME plays a role in PCV3 infection.

### CvME and Macropinocytosis Are Not Involved in PCV3 Infection in PK15 Cells

**FIGURE 3:**
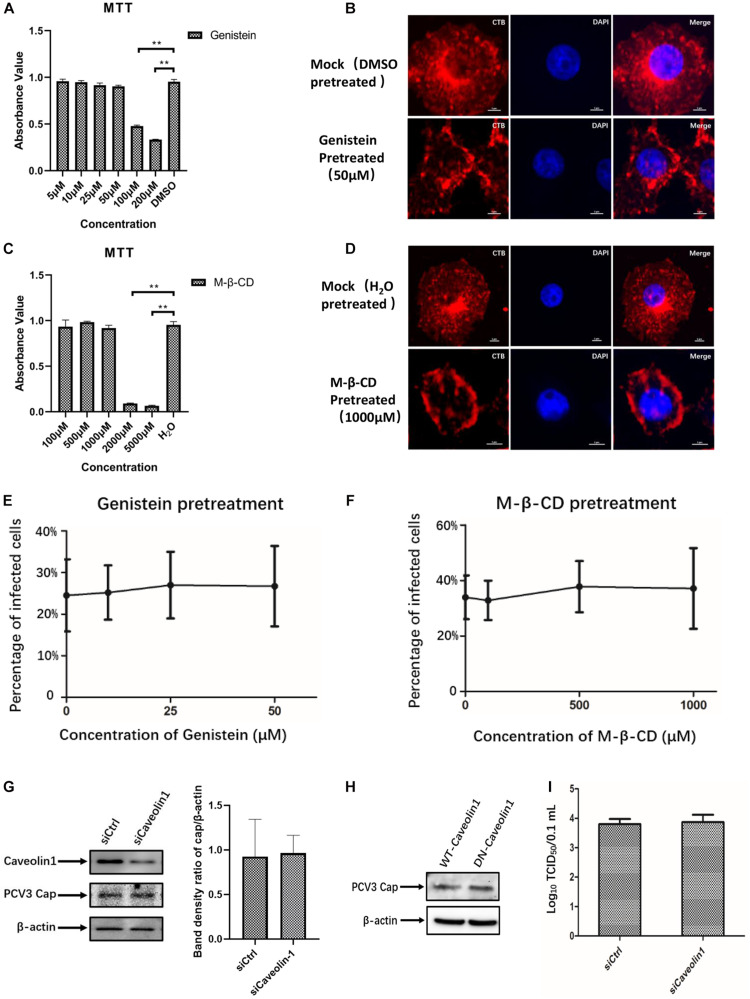
PCV3 infection is independent of caveola-mediated endocytosis. **(A,C)** Cell viability upon genistein or M-β-CD treatment was evaluated by MTT assay. **P < 0.01 indicates statistical significance compared with the mock-treated group. **(B,D)** CT-B uptake in PK15 cells was inhibited by pretreatment with the indicated concentrations of genistein and M-β-CD, which indicated the effective blocking of CvME by both inhibitors. Cells were seeded in chamber slides and exposed to 50 μM genistein **(B)** or 1 mM M-β-CD **(D)** for 1 h at 37°C. Cells treated with the solvent DMSO or ddH_2_O served as the mock groups for genistein and M-β-CD, respectively. Then, 25 μg/mL CT-B-AF647 was added and incubated with the cells in the presence of the corresponding inhibitors for 2 h at 37°C. After washing with PBS to remove the non-attached and non-internalized CTB-AF647 and fixation with 4% PFA, the cells were stained with 4′,6′-diamidino-2-phenylindole (DAPI) for 5 min at RT, mounted, and visualized through immunofluorescence microscopy. Scale bar, 5 μm. **(E,F)** Infection of PCV3 in PK15 cells was not inhibited by genistein or M-β-CD. Cells treated with genistein or M-β-CD at the indicated concentrations or DMSO (mock, 0 μM) and ddH_2_O (mock, 0 μM) were infected with PCV3 for 1 h. After culturing in a medium supplemented with the inhibitors and 2% NBCS for 48 h, cells were fixed and reacted with anti-PCV3 Cap antibodies and observed under immunofluorescence microscopy. The infection rate of PCV3 was assessed as determined by the number of infected cells. **(G)** Cells were transfected with 20 nM siCtrl or 20 nM siCaveolin-1 and then infected with PCV3 for 1 h. After incubated in a medium supplemented with 2% NBCS for 36 h, the knockdown efficiency and viral Cap expression were examined by WB analysis. β-actin served as the loading control for detecting the protein levels. Band density ratio of cap/β-actin was determined for quantitative analysis. **(H)** Caveolin-1-WT or caveolin-1-DN transfected cells were exposed to PCV3 and incubated in medium supplemented with 2% NBCS for 36 h. PCV3 Cap expression was determined by WB. β-actin served as the loading control for detecting the protein levels. **(I)** Cells were transfected with 20 nM siCtrl or 20 nM siCaveolin-1 and then infected with PCV3 and incubated in a medium supplemented with 2% NBCS for 36 h. Then, the cell cultures were harvested and the viral titer was assessed by TCID_50_ after three freeze-thaw cycles.

Given that neither endogenous nor exogenous caveolin-1 was observed to be colocalized with the PCV3 Cap, which may preliminarily exclude the role of caveolin-1 in PCV3 infection, subsequent experiments were conducted to further ascertain the bases of these findings. Genistein, a well-known tyrosine kinase inhibitor that can block the signaling cascade of the CvME pathway, is often used to determine whether viruses enter through the CvME pathway (Lajoie and Nabi, 2007; Li et al., 2017). In addition, CvME is known to be negatively affected by cholesterol depletion caused by chemical perturbation, such as that with M-β-CD (Lee et al., 2008; Mahammad and Parmryd, 2015). In this study, PK15 cells were pretreated with gradient concentrations of genistein or M-β-CD before and during PCV3 infection to study the inhibitory effect of both drugs on viral entry. Cell viability analysis was conducted via MTT assays and a working concentration range of 5–50 and 100–1,000 μM were determined for genistein and M-β-CD, respectively (Figures 3A,C). CT-B was considered to be internalized via lipid rafts/caveolar mediated endocytosis. As a positive control for the effective inhibitory of genistein and M-β-CD, we monitored the uptake of Alexa Fluor 647-conjugated CT-B in PK15 cells pretreated with genistein or M-β-CD. The results showed that in cells pretreated with 50 μM genistein or 1 mM M-β-CD, positive signals for CT-B (red) were mainly located along the cell membrane, whereas in mock (DMSO or ddH_2_O)-treated cells, CT-B was diffusely distributed in the cytoplasm with dense aggregations in areas near the nucleus, which indicated an effective blocking of CvME by both of the inhibitors (Figures 3B,D). However, treating cells with increasing doses of genistein and M-β-CD failed to inhibit PCV3 internalization at a multiplicity of infection (MOI) of 0.5. No significant differences in the number of infected cells were observed between the treated and mock-treated groups (Figures 3E,F). In addition, knockdown of caveolin-1 expression by siRNA did not affect PCV3 Cap expression or viral infectivity compared with those in the siCtrl-transfected groups, as determined by western blotting and the TCID_50_ assay, respectively (Figures 3G,I). Moreover, we transfected the WT or DN constructs of caveolin-1 into PK15 cells prior to PCV3 infection to investigate the impact of overexpression on virus infection. The results showed no obvious difference in the level of PCV3 Cap expression between these two transfected cell groups (Figure 3H). These findings provide evidence that the CvME pathway is not involved in the PCV3 internalization process.

**FIGURE 4:**
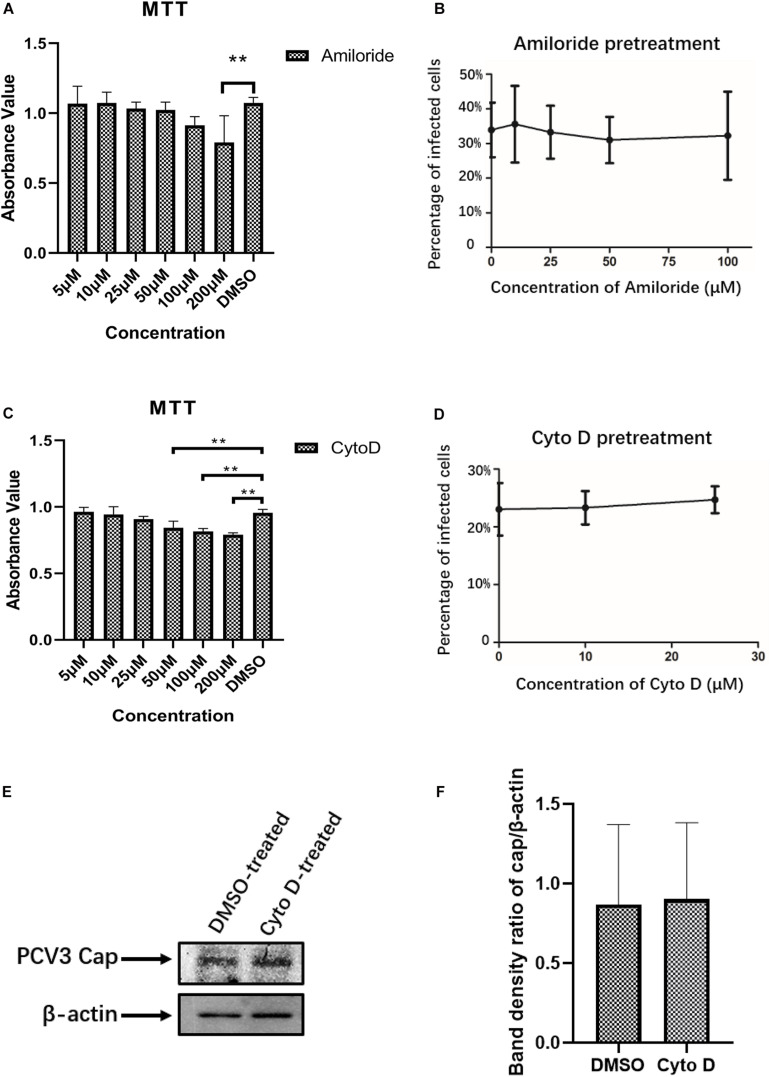
PCV3 infection is independent of macropinocytosis. **(A,C)** Cell viability upon amiloride or Cyto D treatment was evaluated by MTT assay. The results showed no significant cytotoxicity caused by both drugs in the working concentrations in PCV3 infection experiments. **P < 0.01 indicates statistical significance compared with the mock-treated group. **(B)** Infection of PCV3 in PK15 cells was not inhibited by amiloride treatment. Cells treated with amiloride at the indicated concentrations or DMSO (mock, 0 μM) were infected with PCV3 for 1 h. After culturing in a medium in the presence of amiloride or DMSO for 48 h, cells were fixed and reacted with anti-PCV3 Cap antibodies and observed under immunofluorescence microscopy. **(D)** Infection of PCV3 in PK15 cells was not inhibited by Cyto D. Cells pretreated with Cyto D at the indicated concentrations or DMSO (mock, 0 μM) were infected with PCV3 for 1 h. After culturing in a medium supplemented with 2% NBCS for 48 h, the cells were fixed and reacted with anti-PCV3 Cap antibodies and observed under immunofluorescence microscopy. The infection rate of PCV3 was assessed as determined by the number of infected cells. **(E,F)** The results of western blotting showed that PCV3 cap expression in Cyto D-treated cells at a working concentration of 25 μM was not significantly different from that in the mock-treated cell groups. Band density ratio of cap/β-actin was determined for quantitative analysis.

Certain viruses, such as influenza, can exploit different endocytic pathways to gain entry into target cells (Sieczkarski and Whittaker, 2002b; Chen and Zhuang, 2008). To examine the potential involvement of macropinocytosis in PCV3 infection in PK15 cells, increasing doses of amiloride were used to treat the cells prior to and during PCV3 infection. Amiloride is an Na^+^/H^+^ exchanger (NHE) inhibitor that selectively inhibits macropinocytosis-associated plasma membrane blebbing (Koivusalo et al., 2010). The working concentration range of amiloride was determined according to the MTT assay (Figure 4A). Our results showed that although the dosages applied reached as high as 100 μM, amiloride pretreatment had little effect on PCV3 infection, as evidenced by the number of infected cells compared with the mock treatment group (Figure 4B). In addition, since macropinocytosis is also largely dependent on actin-motivated cell membrane ruffling, and Cyto D is able to impede actin polymerization by binding to the ends of F-actin filaments and therefore inhibits cellular macropinocytosis (Brenner and Korn, 1979), we employed increasing doses of Cyto D to treat PK15 cells before and during virus infection. MTT assays were done to analyze cell viability and determine the working concentrations of Cyto D (Figure 4C). PK15 cells were pretreated with gradient concentrations of Cyto D followed by PCV3 infection, and the experimental data showed that this drug did not impair PCV3 infection, as determined by the percentage of infected cells compared with the mock treatment group, as previously described (Figure 4D). The results of western blotting showed that PCV3 cap expression in Cyto D-treated cells at a concentration of 25 μM was not significantly different from that in the mock-treated cell groups (Figures 4E,F). Taken together, our results suggest that macropinocytosis may not serve as a route for entry of PCV3 into PK15 cells.

### Infection of PCV3 in PK15 Cells Is Dynamin-2-Dependent

**FIGURE 5:**
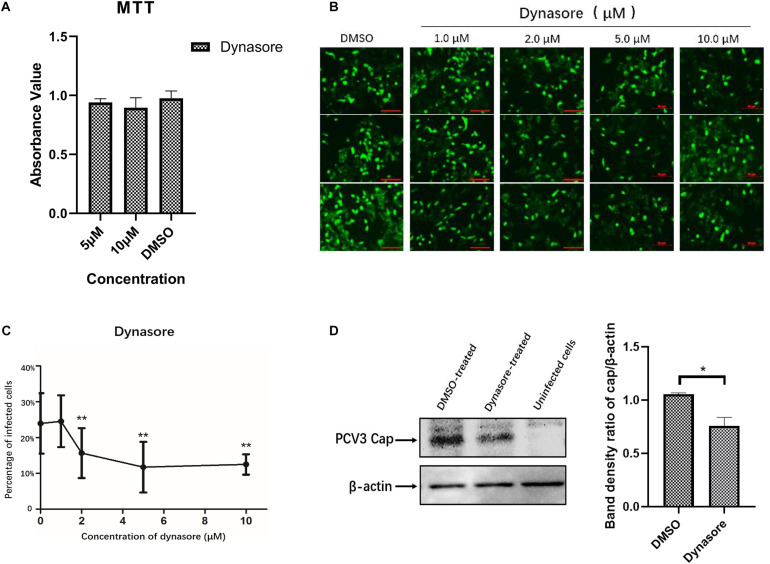
Dynamin-2 is involved in PCV3 infection. **(A)** Cell viability upon dynasore treatment was evaluated via MTT assays. The results showed no significant cytotoxicity caused by dynasore at the working concentrations in PCV3 infection experiments. **(B,C)** Dynasore inhibited PCV3 infection. PK15 cells were exposed to dynasore at the indicated doses or DMSO (mock) for 1 h, and then incubated with PCV3 for 1 h. After culturing in a medium supplemented with dynasore and 2% NBCS for 48 h, the cells were probed with anti-PCV3 Cap antibodies and observed under immunofluorescence microscopy. Scale bar, 50 μm. The infection rate of PCV3 in PK15 cells decreased after dynasore pretreatment in a dose-dependent pattern, as determined by the percentage of infected cells. **P < 0.01 showed significant difference as compared with the mock-treated group (0 μM). **(D)** The results of western blotting showed that PCV3 cap expression was significantly lower in dynasore-treated cells at a working concentration of 10 μM than that in the mock-treated group. Band density ratio of cap/β-actin was determined for quantitative analysis. *P < 0.05 showed significant difference as compared with the mock-treated group.

As the endocytic pits mature and progress toward vesicles, the large GTPase dynamin-2 is recruited to the emanating vesicle neck and helps pinch off the vesicles (Hinshaw, 2000). Dynamin-2 is observed to function in some clathrin-, caveolar-mediated, or CCIP endocytosis of viruses (Gianni et al., 2010; Hernaez and Alonso, 2010). To evaluate the involvement of dynamin-2 in PCV3 infection, PK15 cells were preincubated with the indicated doses of dynasore, a non-competitive inhibitor targeting dynamin GTPase (Macia et al., 2006), prior to and during PCV3 infection. MTT assays demonstrated that no significant cytotoxicity was caused by dynasore at the indicated concentrations used in PCV3 infection experiments (Figure 5A). In this study, PK15 cells were exposed to increasing concentrations of dynasore or DMSO (mock) for 1 h at 37°C, followed by PCV3 infection for 1 h in the presence of dynasore. The cells were then cultured in a medium supplemented with dynasore for 48 h and subjected to IFA assays probing the PCV3 Cap. We observed that the cells treated with dynasore at a dose of 1 μM showed no significant difference compared with the mock-treated group (0 μM) (Figures 5B,C). However, incubation of PK15 cells with 2, 5, and 10 μM dynasore reduced the number of PCV3-infected cells from 24.0% in the untreated group to 15.7, 11.7, and 12.5% in the PCV3-infected cells, respectively, indicating that PCV3 infection was significantly hampered by dynasore treatment in a dose-dependent pattern (Figures 5B,C). The results of western blotting showed that PCV3 cap expression was significantly lower in dynasore-treated cells at a working concentration of 10 μM than that in the mock-treated groups (Figure 5D), suggesting the involvement of dynamin-2 in the PCV3 endocytic process.

### PCV3 Enters the Early Endosomes (EEs) and Late Endosomes (LEs) but Not the Perinuclear Recycling Endosomes (PNREs) During Viral Infection

**FIGURE 6:**
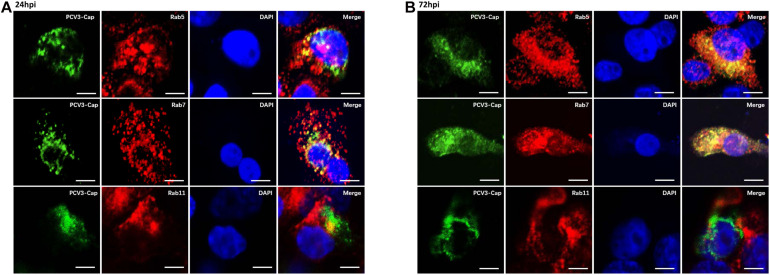
PCV3 Cap colocalized with Rab5 and Rab7, but not Rab11 during PCV3 infection. Cells seeded in chamber slides were incubated with PCV3 and cultured in a medium supplemented with 2% NBCS for 24 h **(A)** or 72 h **(B)** before being fixed at RT for 20 min. Then, the cells were reacted with swine anti-PCV3 Cap (FITC), rabbit anti-Rab5 (TRITC), rabbit anti-Rab7 (TRITC), or rabbit anti-Rab11 antibodies (TRITC) and subjected to immunofluorescence colocalization analysis. Scale bar, 10 μm.

Previous studies have demonstrated that some clathrin-mediated endocytic viruses require endosomal trafficking for successful infection. Classical swine fever virus was observed to enter the EEs and LEs before releasing its RNA in PK15 cells (Shi et al., 2016). EE and PNRE trafficking are reported to be required for the CME pathway during entry of Japanese encephalitis virus into BHK-21 cells (Liu et al., 2017). The Rab family of small GTPases is essential for membrane trafficking and sorting endocytic cargos to specific subcellular compartments (Rink et al., 2005). Rab5, Rab7, and Rab11 are recognized as the dominant regulatory molecules for endocytic cargo sorting and trafficking to the EEs, LEs, and PNREs, respectively, and are the labels for each of the endosomes (Mohrmann and van der Sluijs, 1999; Jordens et al., 2005; Johns et al., 2009). Thus, we continued to determine the colocalization between Cap and Rab proteins to investigate the endosomal trafficking of PCV3. Under confocal microscopic observation, at 24 hpi, the positive signals for Rab5 (red) were found to distribute in the cytoplasm as small spots, and part of the Cap signals (green) were colocalized with Rab5, which are indicated in yellow color (Figure 6A). At 72 hpi, a large amount of colocalization signals for Rab5 and viral Cap were observed to be diffusively distributed in the cytoplasm (Figure 6B). Fluorescence signals for Rab7 (red) also appeared to be punctuate and were distributed in the cytoplasm, and large amounts of its colocalization signals (yellow) with PCV3 Cap were observed at both 24 and 72 hpi (Figures 6A,B). However, the viral Cap was not colocalized with Rab11 either at 24 or 72 hpi (Figures 6A,B). These results suggest that the viral particles travel via EEs and LEs, but not the PNREs after internalization.

### Endosomal Trafficking and an Acidic Environment Are Required for PCV3 Infection

**FIGURE 7:**
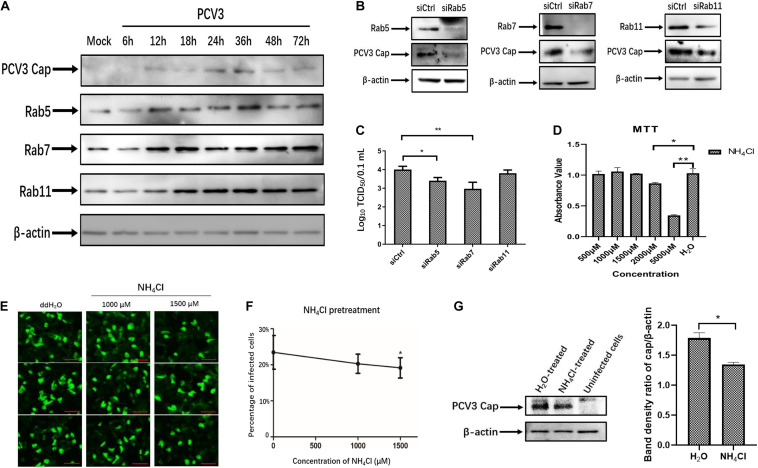
Endosomal trafficking and an acidic environment are required for PCV3 infection. **(A)** Cells were exposed to PCV3 for 1 h and then cultured in a medium supplemented with 2% NBCS for 6, 12, 18, 24, 36, 48, or 72 h. Next, the cells were harvested and the expression of viral Cap, Rab5, Rab7, or Rab11 was examined by WB. Uninfected PK15 cells incubated in a medium supplemented with 2% NBCS for 72 h served as the mock group. **(B)** Cells were transfected with 20 nM siCtrl, 20 nM siRab5, 20 nM siRab7, or 20 nM siRab11 and then infected with PCV3 for 1 h. After incubation in a medium supplemented with 2% NBCS for 36 h, WB analysis was employed to examine the knockdown efficiency and viral Cap expression. β-actin served as the loading control for detecting the protein levels. **(C)** PK15 cells transfected with 20 nM siCtrl, 20 nM siRab5, 20 nM siRab7, or 20 nM siRab11 were infected with PCV3 and incubated in a medium supplemented with 2% NBCS for 36 h. Then, the cell cultures were harvested and the viral titer was assessed by TCID_50_ analysis after three freeze-thaw cycles. *P < 0.05 and **P < 0.01 indicated statistical significance compared with the siCtrl groups. **(D)** Cell viability upon NH_4_Cl treatment was evaluated via MTT assays. *P < 0.05 and **P < 0.01 showed significant difference as compared with the mock-treated group. **(E,F)** NH_4_Cl pretreatment inhibited PCV3 infection in PK15 cells. Cells were incubated with NH_4_Cl at the indicated concentrations or ddH_2_O (mock) for 1 h, then infected with PCV3. After culturing in a medium supplemented with 2% NBCS in the presence of NH_4_Cl for 48 h, the cells were fixed and probed with anti-PCV3 Cap antibodies and examined under immunofluorescence microscopy. Scale bar, 50 μm. The infection rate of PCV3 in PK15 cells decreased after NH_4_Cl treatment in a dose-dependent pattern, as determined by the percentage of infected cells. *P < 0.05 indicate statistical significance compared with the mock treatment group (0 μM). **(G)** The results of western blotting showed that PCV3 cap expression was significantly lower in NH_4_Cl-treated cells at a working concentration of 1,500 μM than that in the mock-treated group. Band density ratio of cap/β-actin was determined for quantitative analysis. *P < 0.05 showed significant difference as compared with the mock-treated group.

The above-described results of PCV3 Cap and Rab colocalization suggest that PCV3 particles may be trafficked to the EEs and LEs for productive infection. Therefore, we examined the regulation of Rab protein expression as well as the role of an acidic environment in PCV3 multiplication in PK15 cells. Initially, we examined the expression of the three Rabs in infected PK15 cells by WB analysis during PCV3 infection. The results showed that the expression levels of Rab5, Rab7, and Rab11 were enhanced in the infected cell groups compared with those in the mock-infected group (Figure 7A). Rab5 expression increased at 12 hpi and reached a peak at 36 hpi, similar to the Cap expression level profile. A higher level of Rab7 was present in the infected cells from 12 to 72 hpi compared with that in mock-infected cells. It is worth noting that Rab11 expression was also markedly enhanced in infected cells from 18 to 72 hpi, despite the colocalization of Rab11 with PCV3 Cap was not observed (Figure 7A). To better determine the role of the three Rab proteins during PCV3 infection, siRNAs were used to knock down Rab expression prior to PCV3 infection. Cells were transfected with 20 nM siCtrl, 20 nM siRab5, 20 nM siRab7, or 20 nM siRab11 before PCV3 infection. At 36 h post PCV3 infection, the level of PCV3 Cap was determined by WB analysis and the viral titers were evaluated by using the TCID_50_ assay. Western blot results demonstrated that PCV3 Cap protein expression was downregulated in siRab5- and siRab7-transfected cells infected with PCV3 compared to that in the siCtrl-transfected cells (Figure 7B). Moreover, the virus titers in both cell cultures decreased significantly after siRab5 and siRab7 transfection compared with those in the siCtrl-transfected groups, as determined by TCID_50_ (Figure 7C). However, the expression level of PCV3 Cap and the virus titers in siRab11-transfected cells were not different from that in the siCtrl-transfected cells (Figures 7B,C).

To examine the effect of an acidic environment on PCV3 infectivity, PK15 cells were incubated with increasing concentrations of NH_4_Cl prior to PCV3 infection. NH_4_Cl is a weak base that functions by inhibiting endosomal acidification (Hemadri and Bandyopadhyay, 1994). MTT assays were employed for cell viability analysis, and the working concentration of NH_4_Cl was determined (Figure 7D). We observed that NH_4_Cl pretreatment significantly reduced the proportion of PCV3-positive cells as determined by IFA (Figures 7E,F). Incubation of cells with 1,500 μM of NH_4_Cl reduced PCV3 infection from 23.5% in the untreated group to 19.2% in the PCV3-infected NH_4_Cl-treated cells (Figures 7E,F). The results of western blotting showed that PCV3 cap expression was significantly lower in NH_4_Cl-treated cells at a working concentration of 1,500 μM than that in the mock-treated groups (Figure 7G), indicating that PCV3 used an endosomal cell entry pathway dependent on a low pH to achieve efficient infection in the cultured cells. Overall, these findings revealed that infection of PCV3 in PK15 cells was regulated by Rab5 and Rab7, and that the virions were sorted into EEs and LEs for efficient infection after internalization.

## Discussion

After being initially reported in the United States in 2015, PCV3 infection has been found in many countries worldwide and has increased as a potential threat to the pig industry (Jiang et al., 2020). Given that PCV3 is a newly discovered virus, increasing investigations have focused on its viral epidemiology, and little information is available regarding its viral entry pathway. The present study was performed to determine the endocytic pathway of PCV3 into the porcine epithelial cell line, PK15 cells. Using double immunofluorescence staining, we found that a large amount of PCV3 virions colocalized with clathrin rather than caveolin-1 after infection (Figures 1A–C). Pharmacological pretreatment with chlorpromazine, knockdown of CLTC expression by RNA interference, or transfection of EPS15-DN significantly impeded viral infection in PK15 cells (Figures 2A–F). Treatment with a dynamin-2 inhibitor, dynasore, also impaired PCV3 infection (Figure 4). These results indicate that clathrin and dynamin-2 are essential for PCV3 infection in PK15 cells. Additionally, we observed the colocalization of PCV3 virus particles with Rab5 and Rab7, but not Rab11, following endocytosis (Figures 6A,B). Knockdown of Rab5 and Rab7 expression by RNA interference or cell pretreatment with ammonium chloride prior to PCV3 addition significantly inhibited viral replication (Figures 7B–F). These findings indicate that PCV3 particles enter the EEs and LEs after internalization, and that viral infection is dependent on Rab5, Rab7, and a low-pH environment. Altogether, our findings demonstrated that PCV3 exploited a clathrin- and dynamin-2-dependent endocytic pathway and the endosomal trafficking system to garner infectious entry into PK15 cells.

Given that the CME and CvME pathways are the most commonly observed endocytic pathway utilized by viruses. In this study, the uptake and localization of the PCV3 was first investigated by laser confocal microscopy to determine whether PCV3 invasion of PK15 cells is related to the CME or CvME pathway. The results showed that a large amount of PCV3 colocalized with clathrin but not caveolin after internalization (Figures 1A–C), suggesting the potential involvement of the CME pathway during PCV3 infection. Clathrin, the key molecule in CME, is an intracellular protein comprised of three heavy- and regulatory light-chain subunits forming a triskelion that can self-assemble into a pentagonal and hexagonal lattice structure, and facilitate invaginated vesicle formation (Popova et al., 2013). In this study, systematic approaches were employed to further assess the contribution of clathrin to PCV3 infection. CPZ is a commonly used drug inhibitor that specifically blocks the CME pathway by impeding clathrin lattice polymerization (Wang et al., 1993). Herein, we found that preincubation of PK15 cells with CPZ significantly impaired PCV3 infection in a dose-dependent manner (Figures 2A–C). Furthermore, knockdown of CLTC by RNA interference reduced viral Cap expression and virus titer *in vitro* (Figures 2D,F). EPS15 functions as a CME-associated adaptor molecule (Gucwa and Brown, 2014). Deletion of the EH domains or overexpression of the C-terminal region leads to a DN effect that inhibits CME, and has been broadly employed to block virus entry through the CME pathway, including bovine viral diarrhea virus (Lecot et al., 2005), classical swine fever virus (Shi et al., 2016), HIV-1 (Daecke et al., 2005), West Nile virus (Chu et al., 2006), and the polyomavirus JC virus (Querbes et al., 2004). Our data showed that the level of PCV3 Cap protein expression in EPS15-DN-transfected cells was obviously lower than that in EPS15-WT-transfected cells as determined by WB assay (Figure 2E). Taken together, our data has demonstrated that PCV3 uses a clathrin-dependent pathway to gain access to PK15 cells, in which the virus can achieve successful infection.

After the clathrin-coated pits formed on the cell membrane where virus-cargo binding takes place, dynamin-2 is recruited and assists in pinching off the maturing pits by self-assembling into a collar structure around the neck area (Hinshaw, 2000). Many viruses have been demonstrated to employ a dynamin-2-clathrin-dependent endocytic pathway to gain access to host cells, such as African swine fever virus (Hernaez and Alonso, 2010), HIV-1 (Daecke et al., 2005), Ebola virus (Aleksandrowicz et al., 2011), and Rift Valley Fever virus (de Boer et al., 2012). Dynamin-2 is proposed to be indispensable and ubiquitously required for the viruses to be internalized in a CME-mediated manner. A previous study demonstrated that entry of PCV2 into epithelial cells is independent of clathrin and dynamin (Misinzo et al., 2009), whereas in our study, PK15 cells pretreated with dynasore, a non-competitive dynamin GTPase activity inhibitor, significantly affected PCV3 infection (Figure 4), which further confirmed the involvement of the CME pathway during PCV3 infection in PK15 cells. Blocking the host pathways essential for the viral life cycle, especially the CME pathway, which is widely used by many viruses, is recognized as an important therapeutic strategy. Baricitinib was reported to disrupt endocytosis, facilitating viral entry by inhibiting AP-2 associated protein kinase 1 and cyclin G- associated kinase (Richardson et al., 2020), both of which are accessory proteins involved in CME. Arbidol is a broad-spectrum antiviral that affects CME of viruses by impeding dynamin-2-triggered membrane scission (Blaising et al., 2013). Sequential host factors associated with the CME of viruses may also serve as effective antiviral targets, and as for PCV3, determination of its host entry pathway may provide insights into the development of antiviral drugs.

Another well-characterized endocytic pathway is the CvME pathway, where endocytic vesicles originate from caveolar abundance with caveolin and cholesterol (Fujimoto et al., 1998). CvME is involved in virus uptake, such as foot-and-mouth disease virus (O’Donnell et al., 2008), simian virus 40 (Pelkmans et al., 2001), and Japanese encephalitis virus (Zhu et al., 2012). In the present study, pharmacological perturbations, including treatments of M-β-CD and genistein, successfully impede CT-B-AF647 internalization via CvME in PK15 cells (Figures 3A–D) but failed to inhibit PCV3 infection (Figures 3E,F), indicating that the CvME pathway was not employed by PCV3 to garner entry into PK15 cells. The results were further verified by immunofluorescence colocalization analysis, siCaveolin1 knockdown, and DN-Caveolin-1 transfection experiments. It is also worth noting that the expression of PCV3 cap in PK15 cells did not decrease but instead increase slightly after siCaveolin-1 transfection or M-β-CD and genistein pretreating. We speculated that interference with the caveolar-dependent endocytic pathway may facilitate the endocytosis of PCV3 to some extent and this deserves further investigation. Pharmacological inhibitors that selectively affect the macropinocytosis process were also used to treat PK15 cells before PCV3 addition, and our data suggest that macropinocytosis is not important for PCV3 infection.

A previous study revealed that PCV2 colocalized with clathrin in epithelial cells, and that inhibiting the CME pathway using CPZ did not impede but instead facilitated viral replication roughly threefold (Misinzo et al., 2009). Moreover, blocking acidification of the endosome-lysosome system also facilitated PCV2 replication in epithelial cells (Misinzo et al., 2008). These findings led to a speculation that PCV2 particles internalized via CME were stuck in the vesicles and were not able to fulfill a replication cycle, which was consistent with the finding that in dendritic cells, PCV2 failed to replicate despite its internalization via CME (Vincent et al., 2003). In the present study, however, we found that successful replication was achieved by PCV3 through the CME pathway in PK15 cells. As far as we know, two kinds of host receptors, the attachment receptor and the uncoating receptor, serve in non-enveloped virus infection (Rossmann et al., 2002; Zhao et al., 2019). After internalization, receptor-mediated capsid uncoating followed by genome release is crucial for viruses to fulfill their life cycle and achieve efficient infection. Although both are known to infect pigs, PCV2 and PCV3 share only a 37% amino acid sequence similarity in their capsid protein (Palinski et al., 2017). We speculate that the different endings of the internalized PCV2 and PCV3 particles via the CME pathway are attributed to the structural differences of these viruses with respect to their capsids. It is possible that the uncoated receptors for PCV3 present in the membrane of endosomal compartments are not recognized by PCV2 due to the difference between the amino acids of their capsids, and may explain why CME of PCV2 does not lead to a productive infection.

Multiple endocytic routes are available for certain viruses to gain access to the same cell type. For example, the influenza virus is capable of gaining access to target cells through clathrin-dependent or clathrin-independent endocytosis (Sieczkarski and Whittaker, 2002b; Chen and Zhuang, 2008). In our study, it is worth noting that both CPZ and dynasore exhibited a marked inhibitory effect on PCV3 entry, but viral infection was not completely prevented. Pretreatment with 2–5 μM of CPZ or dynasore led to a decrease in the number of PCV3-infected cells in a dose-dependent manner, whereas its inhibitory effect approached saturation at a concentration of 10 μM (Figures 2C, 5C). Approximately one-tenth of cells treated with 10 μM CPZ or 10 μM dynasore prior to PCV3 infection is still permissive to the virus, implying the involvement of an unrevealed CCIP in entry of PCV3 into PK15 cells, which merits further exploration.

After detaching from the plasma membrane, endocytic vesicles carrying viruses are transported, sorted, and then fused with other intracellular organelles, such as endosomes. Rabs play a critical role in organizing endocytic cargo sorting and trafficking (Mohrmann and van der Sluijs, 1999). Rab5, predominantly located on EEs, is responsible for coordinating endocytic vesicle fusion with EEs (Bucci et al., 1992). Rab7 mediates traffic from EEs through LEs to lysosomes and is used as a label for LEs (Feng et al., 1995). Meanwhile, PNREs are marked by Rab11 (Sonnichsen et al., 2000). In our study, the endosomal trafficking of PCV3 Cap following internalization was investigated, and the results showed that PCV3 entered the EEs and LEs, but not PNREs, after internalization (Figures 6A,B). The expression of Rab5 and Rab7 increased in the presence of PCV3, and knocking down the expression of both proteins impaired PCV3 infection (Figures 7A–C). This indicates that PCV3 infection is regulated by EE and LE trafficking, which allows for the transport of the viral genome to the preferred subcellular site of replication, and suggests that PCV3 may improve endosomal trafficking efficiency by Rab5 and Rab7 overexpression to facilitate viral transport and proliferation. In addition, endosomal acidification, which is recognized to take place during the process of virus uncoating and genome release, was demonstrated to play a role in PCV3 infection.

In summary, our study demonstrated for the first time that the entry of PCV3 into PK15 cells involves a clathrin- and dynamin-2-mediated endocytic pathway, where early and late endosomal trafficking, as well as an acidic environment, are essential for viral infection. Our findings may pave the way toward a better understanding of the PCV3 life cycle and pathogenesis, and provide new insights into finding new antiviral targets and drugs against PCV3 infection.

## Data Availability Statement

The original contributions presented in the study are included in the article/supplementary material, further inquiries can be directed to the corresponding author/s.

## Author Contributions

JL and RS designed the experiments and drafted the article. RS, LH, and RQ were responsible for performing most of the experiments. LW and JW cultured the cells and viruses. HJ, SZ, JS, and DW were responsible for the statistical analysis of the data. JL and BZ revised the article. All authors have read and approved the final manuscript.

## Conflict of Interest

The authors declare that the research was conducted in the absence of any commercial or financial relationships that could be construed as a potential conflict of interest.

## References

[B1] Aleksandrowicz P. Marzi A. Biedenkopf N. Beimforde N. Becker S. Hoenen T. (2011). Ebola virus enters host cells by macropinocytosis and clathrin-mediated endocytosis. *J. Infect. Dis.* 204(Suppl. 3) S957–S967. 10.1093/infdis/jir326 21987776 PMC3189988

[B2] Benmerah A. Bayrou M. Cerf-Bensussan N. Dautry-Varsat A. (1999). Inhibition of clathrin-coated pit assembly by an Eps15 mutant. *J. Cell Sci.* 112(Pt 9) 1303–1311. 10194409 10.1242/jcs.112.9.1303

[B3] Blaising J. Lévy P. L. Polyak S. J. Stanifer M. Boulant S. Pécheur E. I. (2013). Arbidol inhibits viral entry by interfering with clathrin-dependent trafficking. *Antiviral. Res.* 100 215–219. 10.1016/j.antiviral.2013.08.008 23981392

[B4] Boer S. M. Kortekaas J. Spel L. Rottier P. J. Moormann R. J. Bosch B. J. (2012). Acid-activated structural reorganization of the Rift Valley fever virus Gc fusion protein. *J. Virol.* 86 13642–13652. 10.1128/JVI.01973-12 23035232 PMC3503025

[B5] Brenner S. L. Korn E. D. (1979). Substoichiometric concentrations of cytochalasin D inhibit actin polymerization. Additional evidence for an F-actin treadmill. *J. Biol. Chem.* 254 9982–9985. 489616

[B6] Bucci C. Parton R. G. Mather I. H. Stunnenberg H. Simons K. Hoflack B. (1992). The small GTPase rab5 functions as a regulatory factor in the early endocytic pathway. *Cell* 70 715–728. 10.1016/0092-8674(92)90306-W 1516130

[B7] Cardin A. D. Weintraub H. J. (1989). Molecular modeling of protein-glycosaminoglycan interactions. *Arteriosclerosis* 9 21–32. 10.1161/01.ATV.9.1.21 2463827

[B8] Chen C. Zhuang X. (2008). Epsin 1 is a cargo-specific adaptor for the clathrin-mediated endocytosis of the influenza virus. *Proc. Natl. Acad. Sci. U.S.A.* 105 11790–11795. 10.1073/pnas.0803711105 18689690 PMC2504482

[B9] Chen G. H. Mai K. J. Zhou L. Wu R. T. Tang X. Y. Wu J. L. (2017). Detection and genome sequencing of porcine circovirus 3 in neonatal pigs with congenital tremors in South China. *Transbound Emerg. Dis.* 64 1650–1654. 10.1111/tbed.12702 28975769

[B10] Cheung A. K. (2003). Comparative analysis of the transcriptional patterns of pathogenic and nonpathogenic porcine circoviruses. *Virology* 310 41–49. 10.1016/S0042-6822(03)00096-5 12788629

[B11] Chu J. J. Leong P. W. Ng M. L. (2006). Analysis of the endocytic pathway mediating the infectious entry of mosquito-borne flavivirus West Nile into Aedes albopictus mosquito (C6/36) cells. *Virology* 349 463–475. 10.1016/j.virol.2006.01.022 16490225

[B12] Daecke J. Fackler O. T. Dittmar M. T. Krausslich H. G. (2005). Involvement of clathrin-mediated endocytosis in human immunodeficiency virus type 1 entry. *J. Virol.* 79 1581–1594. 10.1128/JVI.79.3.1581-1594.2005 15650184 PMC544101

[B13] Ellis J. (2014). Porcine circovirus: a historical perspective. *Vet. Pathol.* 51 315–327. 24569612 10.1177/0300985814521245

[B14] Fenaux M. Halbur P. G. Haqshenas G. Royer R. Thomas P. Nawagitgul P. (2002). Cloned genomic DNA of type 2 porcine circovirus is infectious when injected directly into the liver and lymph nodes of pigs: characterization of clinical disease, virus distribution, and pathologic lesions. *J. Virol.* 76 541–551. 10.1128/JVI.76.2.541-551.2002 11752145 PMC136831

[B15] Feng Y. Press B. Wandinger-Ness A. (1995). Rab 7: an important regulator of late endocytic membrane traffic. *J. Cell Biol.* 131(6 Pt 1) 1435–1452. 10.1083/jcb.131.6.1435 8522602 PMC2120682

[B16] Fujimoto T. Hagiwara H. Aoki T. Kogo H. Nomura R. (1998). Caveolae: from a morphological point of view. *J. Electron. Microsc. (Tokyo)* 47 451–460. 10.1093/oxfordjournals.jmicro.a023616 9881454

[B17] Gauger P. C. Lager K. M. Vincent A. L. Opriessnig T. Cheung A. K. Butler J. E. (2011). Leukogram abnormalities in gnotobiotic pigs infected with porcine circovirus type 2. *Vet. Microbiol.* 154 185–190. 10.1016/j.vetmic.2011.06.016 21784586

[B18] Gianni T. Gatta V. Campadelli-Fiume G. (2010). {alpha}V{beta}3-integrin routes herpes simplex virus to an entry pathway dependent on cholesterol-rich lipid rafts and dynamin2. *Proc. Natl. Acad. Sci. U.S.A.* 107 22260–22265. 10.1073/pnas.1014923108 21135248 PMC3009828

[B19] Gresham A. Giles N. Weaver J. (2000). PMWS and porcine dermatitis nephropathy syndrome in Great Britain. *Vet. Rec.* 147:115. 10.1016/S0304-4017(00)00267-3 10955884

[B20] Gucwa A. L. Brown D. A. (2014). UIM domain-dependent recruitment of the endocytic adaptor protein Eps15 to ubiquitin-enriched endosomes. *BMC Cell Biol.* 15:34. 10.1186/1471-2121-15-34 25260758 PMC4181756

[B21] Helenius A. (2018). Virus entry: looking back and moving forward. *J. Mol. Biol.* 430 1853–1862. 10.1016/j.jmb.2018.03.034 29709571 PMC7094621

[B22] Hemadri D. Bandyopadhyay S. K. (1994). Effect of ammonium chloride on multiplication of rinderpest virus in Vero cells. *Acta Virol.* 38 163–167. 10.1016/0168-1702(94)90019-1 7817898

[B23] Hernaez B. Alonso C. (2010). Dynamin- and clathrin-dependent endocytosis in African swine fever virus entry. *J. Virol.* 84 2100–2109. 10.1128/JVI.01557-09 19939916 PMC2812401

[B24] Hinshaw J. E. (2000). Dynamin and its role in membrane fission. *Annu. Rev. Cell Dev. Biol.* 16 483–519. 10.1146/annurev.cellbio.16.1.483 11031245 PMC4781412

[B25] Jiang H. Wang D. Wang J. Zhu S. She R. Ren X. (2019). Induction of porcine dermatitis and nephropathy syndrome in piglets by infection with porcine circovirus type 3. *J. Virol.* 93:e02045–18. 10.1128/JVI.02045-18 30487279 PMC6363995

[B26] Jiang H. Wei L. Wang D. Wang J. Zhu S. She R. (2020). ITRAQ-based quantitative proteomics reveals the first proteome profiles of piglets infected with porcine circovirus type 3. *J. Proteomics* 212:103598. 10.1016/j.jprot.2019.103598 31785380

[B27] Johns H. L. Berryman S. Monaghan P. Belsham G. J. Jackson T. (2009). A dominant-negative mutant of rab5 inhibits infection of cells by foot-and-mouth disease virus: implications for virus entry. *J. Virol.* 83 6247–6256. 10.1128/JVI.02460-08 19357169 PMC2687373

[B28] Jordens I. Marsman M. Kuijl C. Neefjes J. (2005). Rab proteins, connecting transport and vesicle fusion. *Traffic* 6 1070–1077. 10.1111/j.1600-0854.2005.00336.x 16262719

[B29] Koivusalo M. Welch C. Hayashi H. Scott C. C. Kim M. Alexander T. (2010). Amiloride inhibits macropinocytosis by lowering submembranous pH and preventing Rac1 and Cdc42 signaling. *J. Cell Biol.* 188 547–563. 10.1083/jcb.200908086 20156964 PMC2828922

[B30] Krakowka S. Hartunian C. Hamberg A. Shoup D. Rings M. Zhang Y. (2008). Evaluation of induction of porcine dermatitis and nephropathy syndrome in gnotobiotic pigs with negative results for porcine circovirus type 2. *Am. J. Vet. Res.* 69 1615–1622. 10.2460/ajvr.69.12.1615 19046009

[B31] Lajoie P. Nabi I. R. (2007). Regulation of raft-dependent endocytosis. *J. Cell Mol. Med.* 11 644–653. 10.1111/j.1582-4934.2007.00083.x 17760830 PMC3823247

[B32] Lecot S. Belouzard S. Dubuisson J. Rouille Y. (2005). Bovine viral diarrhea virus entry is dependent on clathrin-mediated endocytosis. *J. Virol.* 79 10826–10829. 10.1128/JVI.79.16.10826-10829.2005 16051874 PMC1182683

[B33] Lee C. J. Lin H. R. Liao C. L. Lin Y. L. (2008). Cholesterol effectively blocks entry of flavivirus. *J. Virol.* 82 6470–6480. 10.1128/JVI.00117-08 18448543 PMC2447114

[B34] Li Z. Zhao K. Lan Y. Lv X. Hu S. Guan J. (2017). Porcine hemagglutinating encephalomyelitis virus enters Neuro-2a cells via clathrin-mediated endocytosis in a Rab5-, Cholesterol-, and pH-Dependent Manner. *J. Virol.* 91:e01083–17. 10.1128/JVI.01083-17 28956766 PMC5686734

[B35] Liu C. C. Zhang Y. N. Li Z. Y. Hou J. X. Zhou J. Kan L. (2017). Rab5 and Rab11 are required for clathrin-dependent endocytosis of Japanese encephalitis virus in BHK-21 cells. *J. Virol.* 91:e01113–17. 10.1128/JVI.01113-17 28724764 PMC5599755

[B36] Macia E. Ehrlich M. Massol R. Boucrot E. Brunner C. Kirchhausen T. (2006). Dynasore, a cell-permeable inhibitor of dynamin. *Dev. Cell* 10 839–850. 10.1016/j.devcel.2006.04.002 16740485

[B37] Mahammad S. Parmryd I. (2015). Cholesterol depletion using methyl-beta-cyclodextrin. *Methods Mol. Biol.* 1232 91–102. 10.1007/978-1-4939-1752-5_8 25331130

[B38] Mercer J. Schelhaas M. Helenius A. (2010). Virus entry by endocytosis. *Annu. Rev. Biochem.* 79 803–833. 10.1146/annurev-biochem-060208-104626 20196649

[B39] Misinzo G. Delputte P. L. Lefebvre D. J. Nauwynck H. J. (2009). Porcine circovirus 2 infection of epithelial cells is clathrin-, caveolae- and dynamin-independent, actin and Rho-GTPase-mediated, and enhanced by cholesterol depletion. *Virus Res.* 139 1–9. 10.1016/j.virusres.2008.09.005 18952130

[B40] Misinzo G. Delputte P. L. Meerts P. Lefebvre D. J. Nauwynck H. J. (2006). Porcine circovirus 2 uses heparan sulfate and chondroitin sulfate B glycosaminoglycans as receptors for its attachment to host cells. *J. Virol.* 80 3487–3494. 10.1128/JVI.80.7.3487-3494.2006 16537616 PMC1440380

[B41] Misinzo G. Delputte P. L. Nauwynck H. J. (2008). Inhibition of endosome-lysosome system acidification enhances porcine circovirus 2 infection of porcine epithelial cells. *J. Virol.* 82 1128–1135. 10.1128/JVI.01229-07 18032516 PMC2224462

[B42] Misinzo G. Meerts P. Bublot M. Mast J. Weingartl H. M. Nauwynck H. J. (2005). Binding and entry characteristics of porcine circovirus 2 in cells of the porcine monocytic line 3D4/31. *J. Gen. Virol.* 86(Pt 7) 2057–2068. 10.1099/vir.0.80652-0 15958685

[B43] Mohrmann K. van der Sluijs P. (1999). Regulation of membrane transport through the endocytic pathway by rabGTPases. *Mol. Membr. Biol.* 16 81–87. 10.1080/096876899294797 10332741

[B44] O’Donnell V. Larocco M. Baxt B. (2008). Heparan sulfate-binding foot-and-mouth disease virus enters cells via caveola-mediated endocytosis. *J. Virol.* 82 9075–9085. 10.1128/JVI.00732-08 18614639 PMC2546884

[B45] Oh T. Chae C. (2020). First isolation and genetic characterization of porcine circovirus type 3 using primary porcine kidney cells. *Vet. Microbiol.* 241:108576. 10.1016/j.vetmic.2020.108576 31928694

[B46] Opriessnig T. Langohr I. (2013). Current state of knowledge on porcine circovirus type 2-associated lesions. *Vet. Pathol.* 50 23–38. 10.1177/0300985812450726 22692624

[B47] Palinski R. Pineyro P. Shang P. Yuan F. Guo R. Fang Y. (2017). A novel porcine circovirus distantly related to known circoviruses is associated with porcine dermatitis and nephropathy syndrome and reproductive failure. *J. Virol.* 91:e01879–16. 10.1128/JVI.01879-16 27795441 PMC5165205

[B48] Pelkmans L. Helenius A. (2002). Endocytosis via caveolae. *Traffic* 3 311–320. 10.1034/j.1600-0854.2002.30501.x 11967125

[B49] Pelkmans L. Kartenbeck J. Helenius A. (2001). Caveolar endocytosis of simian virus 40 reveals a new two-step vesicular-transport pathway to the ER. *Nat. Cell Biol.* 3 473–483. 10.1038/35074539 11331875

[B50] Phan T. G. Giannitti F. Rossow S. Marthaler D. Knutson T. P. Li L. (2016). Detection of a novel circovirus PCV3 in pigs with cardiac and multi-systemic inflammation. *Virol. J.* 13:184. 10.1186/s12985-016-0642-z 27835942 PMC5105309

[B51] Popova N. V. Deyev I. E. Petrenko A. G. (2013). Clathrin-mediated endocytosis and adaptor proteins. *Acta Nat.* 5 62–73. 10.2119/molmed.2013.00108 24307937 PMC3848845

[B52] Querbes W. Benmerah A. Tosoni D. Di Fiore P. P. Atwood W. J. (2004). A JC virus-induced signal is required for infection of glial cells by a clathrin- and eps15-dependent pathway. *J. Virol.* 78 250–256. 10.1128/JVI.78.1.250-256.2004 14671106 PMC303400

[B53] Richardson P. Griffin I. Tucker C. Smith D. Oechsle O. Phelan A. (2020). Baricitinib as potential treatment for 2019-nCoV acute respiratory disease. *Lancet* 395 e30–e31. 10.1016/S0140-6736(20)30304-4 32032529 PMC7137985

[B54] Rink J. Ghigo E. Kalaidzidis Y. Zerial M. (2005). Rab conversion as a mechanism of progression from early to late endosomes. *Cell* 122 735–749. 10.1016/j.cell.2005.06.043 16143105

[B55] Rose N. Opriessnig T. Grasland B. Jestin A. (2012). Epidemiology and transmission of porcine circovirus type 2 (PCV2). *Virus Res.* 164 78–89. 10.1016/j.virusres.2011.12.002 22178804

[B56] Rossmann M. G. He Y. Kuhn R. J. (2002). Picornavirus-receptor interactions. *Trends Microbiol.* 10 324–331. 10.1016/S0966-842X(02)02383-1 12110211

[B57] Shi B. J. Liu C. C. Zhou J. Wang S. Q. Gao Z. C. Zhang X. M. (2016). Entry of classical swine fever virus into PK-15 Cells via a pH-, dynamin-, and cholesterol-dependent, clathrin-mediated endocytic pathway that requires Rab5 and Rab7. *J. Virol.* 90 9194–9208. 10.1128/JVI.00688-16 27489278 PMC5044825

[B58] Sieczkarski S. B. Whittaker G. R. (2002a). Dissecting virus entry via endocytosis. *J. Gen. Virol.* 83(Pt 7) 1535–1545. 10.1099/0022-1317-83-7-1535 12075072

[B59] Sieczkarski S. B. Whittaker G. R. (2002b). Influenza virus can enter and infect cells in the absence of clathrin-mediated endocytosis. *J. Virol.* 76 10455–10464. 10.1128/JVI.76.20.10455-10464.2002 12239322 PMC136567

[B60] Sonnichsen B. De Renzis S. Nielsen E. Rietdorf J. Zerial M. (2000). Distinct membrane domains on endosomes in the recycling pathway visualized by multicolor imaging of Rab4. Rab5, and Rab11. *J. Cell Biol.* 149 901–914. 10.1083/jcb.149.4.901 10811830 PMC2174575

[B61] Takei K. Haucke V. (2001). Clathrin-mediated endocytosis: membrane factors pull the trigger. *Trends Cell Biol.* 11 385–391. 10.1016/S0962-8924(01)02082-7 11514193

[B62] Tischer I. Mields W. Wolff D. Vagt M. Griem W. (1986). Studies on epidemiology and pathogenicity of porcine circovirus. *Arch. Virol.* 91 271–276. 10.1007/BF01314286 3778212

[B63] Vincent I. E. Carrasco C. P. Herrmann B. Meehan B. M. Allan G. M. Summerfield A. (2003). Dendritic cells harbor infectious porcine circovirus type 2 in the absence of apparent cell modulation or replication of the virus. *J. Virol.* 77 13288–13300. 10.1128/JVI.77.24.13288-13300.2003 14645585 PMC296043

[B64] Wang L. H. Rothberg K. G. Anderson R. G. (1993). Mis-assembly of clathrin lattices on endosomes reveals a regulatory switch for coated pit formation. *J. Cell Biol.* 123 1107–1117. 10.1083/jcb.123.5.1107 8245121 PMC2119875

[B65] Wei L. Kwang J. Wang J. Shi L. Yang B. Li Y. (2008). Porcine circovirus type 2 induces the activation of nuclear factor kappa B by IkappaBalpha degradation. *Virology* 378 177–184. 10.1016/j.virol.2008.05.013 18561971

[B66] Zhai S. L. Chen S. N. Xu Z. H. Tang M. H. Wang F. G. Li X. J. (2014). Porcine circovirus type 2 in China: an update on and insights to its prevalence and control. *Virol. J.* 11:88. 10.1186/1743-422X-11-88 24885983 PMC4031328

[B67] Zhao X. Zhang G. Liu S. Chen X. Peng R. Dai L. (2019). Human neonatal fc receptor is the cellular uncoating receptor for enterovirus B. *Cell, 177(6)* 155:e1516. 10.1016/j.cell.2019.04.035 31104841 PMC7111318

[B68] Zhu Y. Z. Xu Q. Q. Wu D. G. Ren H. Zhao P. Lao W. G. (2012). Japanese encephalitis virus enters rat neuroblastoma cells via a pH-dependent, dynamin and caveola-mediated endocytosis pathway. *J. Virol.* 86 13407–13422. 10.1128/JVI.00903-12 23015720 PMC3503063

